# Functional Nanomaterials for Sensing and Detection (2nd Edition)

**DOI:** 10.3390/nano15080588

**Published:** 2025-04-11

**Authors:** Weiping Cai, Hongwen Zhang

**Affiliations:** Key Laboratory of Materials Physics, Anhui Key Laboratory of Nanomaterials and Nanotechnology, Institute of Solid State Physics, HFIPS, Chinese Academy of Sciences, Hefei 230031, China; hwzhang@issp.ac.cn

Functional nanomaterials have emerged as a cornerstone of modern sensing and detection technologies, owing to their unique physicochemical properties derived from high surface-to-volume ratios and nanoscale effects. These materials, spanning zero-dimensional, one-dimensional, and two-dimensional architectures—such as nanoparticles, nanowires, and nanosheets—exhibit exceptional performance in applications ranging from environmental monitoring to biomedical diagnostics. Their versatility is further amplified by their roles as transducers, signal amplifiers, and capture agents in devices like chemiresistive sensors and surface-enhanced Raman spectroscopy (SERS) platforms. This Special Issue, Functional Nanomaterials for Sensing and Detection (2nd Edition), collates seven cutting-edge studies that exemplify recent advancements in the design, fabrication, and application of these materials. The contributions herein highlight innovations in magnetic nanoparticle manipulation, plasmonic nanostructures, hybrid nanocomposites, and metasurface technologies, collectively addressing critical challenges in sensitivity, selectivity, and real-world deployability.

The seven articles featured in this Special Issue span a diverse array of nanomaterials and sensing techniques. Dowling and Kostylev demonstrate the controlled capture of magnetic nanoparticles (MNPs) using ferromagnetic antidot and dot nanostructures under microfluidic conditions [1]. By applying parallel or perpendicular magnetic fields, they achieved up to 84% capture efficiency, showcasing potential for high-sensitivity biosensing and filtration applications. This work underscores the importance of magnetic field engineering in optimizing MNP distribution for detection. Mo et al. developed a Au-ordered array substrate for the rapid SERS detection of etomidate in e-liquids [2]. Their substrate, featuring uniform electromagnetic hotspots, enabled the sensitive identification of trace analytes in complex matrices, offering a promising tool for the on-site screening of illicit substances. The integration of spectral reproducibility and quantitative precision positions this technology as a bridge between laboratory analysis and real-world deployment.

Hatsuoka et al. explored tunable plasmon resonance in silver nanodisk-on-mirror structures, achieving a fivefold enhancement in scattering intensity through annealing [3]. Their finite-difference time-domain simulations and experimental validations revealed the critical role of spacer-layer thickness and nanostructure geometry in tailoring plasmonic responses, with implications for optical sensing and emission enhancement. Similarly, Ahmad et al. engineered an enzymeless α-Fe_2_O_3_-ZnO hybrid sensor for nitrite detection, achieving a remarkable sensitivity of 18.10 µA µM^−1^ cm^−2^ and a low detection limit of 0.16 µM [4]. The hybrid nanostructure’s stability and selectivity in serum samples highlight its potential for environmental and clinical monitoring.

Liu et al. reported imine-linked covalent organic framework nanospheres (COF) for ethylene glycol sensing, achieving a 40 ppb detection limit via mesoporous structures and hydrogen bonding interactions [5]. The material’s selectivity against 20 interfering gases underscores the value of defect engineering and surface functionalization in gas sensing. Iwanaga proposed a rational design strategy for refractive-index sensors using plasmonic lattice structures and silicon metasurfaces, emphasizing dual-parameter (wavelength and amplitude) sensing to approach physical performance limits [6]. Finally, Liu et al. designed a pixelated metasurface filter array for on-chip polarized spectral detection, achieving 75% transmission efficiency and 10 nm resolution in the near-infrared range [7]. This complementary metal–oxide–semiconductor (CMOS)-compatible platform paves the way for compact, high-efficiency spectral imaging systems.

Collectively, these studies exemplify the interdisciplinary synergy between material science, nanotechnology, and device engineering. However, challenges remain in scalability, long-term stability under real-world conditions, and cost-effective fabrication. For instance, while the Au SERS substrate [2] offers exceptional sensitivity, its large-scale production and integration into portable devices warrant further exploration. Similarly, the COF-based sensor [5] necessitates validation in humid or variable-temperature environments to assess practical viability.

In summary, this Special Issue underscores the transformative potential of functional nanomaterials in advancing sensing and detection technologies. The featured works highlight innovative strategies to enhance sensitivity, selectivity, and multiplexing capabilities, from magnetic field-guided nanoparticle capture to metasurface-enabled spectral resolution. Looking ahead, several directions merit attention: (1) the development of multifunctional nanocomposites for the simultaneous detection of multiple analytes; (2) the integration of machine learning algorithms with sensor data to improve pattern recognition in complex matrices; and (3) the exploration of sustainable, bio-compatible nanomaterials for in vivo or environmental applications. Furthermore, bridging the gap between laboratory prototypes and commercial devices will require collaboration across disciplines, including microfabrication, electronics, and data science. With the rapid evolution of the field, functional nanomaterials are set to revolutionize sensing technology, paving the way for smarter, faster, and more accessible solutions that address critical challenges in global health, safety, and environmental sustainability.
